# Effect and safety of acupuncture for *Hwa-byung*, an anger syndrome: a study protocol of a randomized controlled pilot trial

**DOI:** 10.1186/s13063-017-2399-0

**Published:** 2018-02-09

**Authors:** Hye-Yoon Lee, Jung-Eun Kim, Mikyung Kim, Ae-Ran Kim, Hyo-Ju Park, O-Jin Kwon, Jung-Hyo Cho, Sun-Yong Chung, Joo-Hee Kim

**Affiliations:** 1 Research Institute for Korean Medicine, Pusan National University, 20, Geumo-ro, Mulgeum, Yangsan Republic of Korea; 2 Clinical Research Division, Korea Institute of Oriental Medicine, 1672, Yuseong-daero, Yuseong-gu, Daejeon Republic of Korea; 3 Department of Internal Medicine, College of Korean Medicine, Sangji University, 83, Sangjidae-gil, Wonju, Republic of Korea; 4 Department of Internal Korean Medicine, Daejeon Oriental Hospital of Daejeon University, 175, Daeheung-ro, Jung-gu, Daejeon Republic of Korea; 5 Department of Neuropsychiatry, College of Korean Medicine, Kyung Hee University, 892, Dongnam-ro, Gangdong-gu, Seoul Republic of Korea; 6 Department of Acupuncture and Moxibustion Medicine, College of Korean Medicine, Sangji University, 83, Sangjidae-gil, Wonju-si, Gangwon-do Republic of Korea

**Keywords:** *Hwa-byung*, Anger syndrome, Acupuncture, Psychiatric disorder, Randomized controlled trial

## Abstract

**Background:**

*Hwa-byung* (HB) is an anger syndrome caused by an inadequate release of accumulated anger that leads to somatic and psychiatric symptoms. As HB results from long-term inadequately treated negative emotions, its symptoms are complex, intractable and concomitant with other psychiatric disorders. Therefore, studies aiming to develop effective and safe treatment options for HB are needed. We plan to conduct a pilot study for a future, full-scale, randomized controlled trial (RCT) of an optimal acupuncture procedure using semi-individualized acupuncture points that consider participants’ personal disposition and type of emotional stress.

**Method/design:**

This randomized, sham-controlled, participant- and assessor-blinded pilot trial aims to determine the study feasibility of acupuncture for HB and to explore its clinical effects and safety. This clinical trial will be conducted with two groups: one treated with real acupuncture and the other with sham acupuncture for 10 sessions over 4 weeks. The experimental group (EG) will receive semi-individualized acupuncture, whereas the control group (CG) will receive sham acupuncture, namely minimal acupuncture on non-acupuncture points. The recruitment, compliance, and completion rate and clinical evaluations, including a Visual Analogue Scale (VAS), the Korean version of the Beck Depression Inventory (BDI), the short form of the Stress Response Inventory (SRI-short form) and the Instrument of the Oriental Medical Evaluation for HB (IOME-HB), will be assessed to evaluate feasibility and possible effects and safety. Four weeks after completing treatment, follow-up assessments will be performed.

**Discussion:**

As this is a pilot study mainly aiming to investigate trial feasibility, the results of this study will be analyzed descriptively and interpreted for the study purposes. Cohen’s *d* will be reported to determine the effect of acupuncture for HB and to enable comparisons with other treatment methods. This protocol is significant in that it provides optimal semi-individualized acupuncture treatment. We expect this study to offer information about the feasibility of this treatment and data about the possible effects and safety.

**Trial registration:**

Clinical Research Information Service (CRIS), Republic of Korea: KCT0001732. Registered on 14 December 2015.

**Electronic supplementary material:**

The online version of this article (doi:10.1186/s13063-017-2399-0) contains supplementary material, which is available to authorized users.

## Background

*Hwa-byung* (HB) is an anger syndrome that is considered a cultural syndrome of Korea. In the *Diagnostic and Statistical Manual of Mental Disorders, version 4* (DSM-IV), HB is listed as a Korean culture-bound syndrome in the glossary of culture-bound syndromes [1], and the disease has been given an independent disease code in the *Korean Standard Classification of Diseases 7* (KCD-7) [2]. Research is currently underway to diagnose HB separately from other mental disorders, as diagnostic criteria change with the culture of the time. Accordingly, the HB Diagnostic Interview Schedule (HBDIS) and the Instrument of Oriental Medical Evaluation for HB (IOME-HB) were developed [3–5]. HB has distinct characteristics that differ from those of other known psychiatric disorders, and is typically accompanied by major depressive disorder (MDD) and generalized anxiety disorder (GAD) [6–8].

The symptoms of HB can be physical and include chest palpitations, flushing, surges of feelings, and lumps in the throat or solar plexus; however, they can also be emotional and include resentment, bitterness, and hard feelings [3]. HB results from anger that has accumulated over a long period of time without being released and is caused both by an individual’s disposition and by repeated stress from a society that imposes unfair and unreasonable restrictions on its members [3, 6, 7]. The prevalence of HB ranges from 4.2% to 13.3% in Korea depending on the age group and region [3, 9] and is reported to be higher in women than in men [10]. Self-perceived HB is relatively prevalent, with a community-based study reporting a rate of 12% [11].

One review study indicated that a multi-disciplinary approach, including religious help and traditional methods, is necessary for the treatment of HB [12]. Another study investigating paroxetine revealed improvements in the symptoms of HB and Hamilton Depression Rating Scale (HAM-D) and State-Trait Anger Expression Inventory (STAXI) scores, but the study was limited to a single-group, open trial [13]. In terms of non-pharmacological treatments, one qualitative study investigating loving-kindness meditation reported therapeutic effects in four of nine participants, partial effects in four participants, and no effect in one participant [14]. Another qualitative study investigating a mindfulness-based stress reduction program indicated improvement in the symptoms of HB and STAXI, Hospital Anxiety and Depression Scale-Anxiety (HAD-A), and Hospital Anxiety and Depression scale-Depression (HAD-D) score [15]; however, these two studies included small sample sizes (9 and 10 participants, respectively) and were single-arm, qualitative studies whose findings are difficult to generalize.

Acupuncture has been used for various psychiatric disorders [16], and its clinical effect against depression [17] and anxiety [18, 19] has been reported in previous studies. For studies on HB, one study reported improvement in the symptoms of HB and Beck Depression Inventory (BDI) score but not in STAXI and State-Trait Anxiety Inventory (STAI) scores [20]. Several studies reported contrasting results, and did not find significant improvement in symptoms of HB or in BDI, STAXI, or STAI scores [21, 22]. These three studies included a control group that received shallow, penetrating, sham acupuncture at non-acupuncture points, which may have contributed to a failure to detect differences between the groups. In addition, the acupuncture used in these studies was focused on traditional eastern philosophy using the Five Elements Theory; thus, the method exhibited limitations in the possible widespread use by global researchers and practitioners. Two studies focused on concurrent symptoms, such as insomnia and anxiety, but remained limited in that one used a waiting list and the other included a single-group, open-trial design [23]. In 2013, clinical guidelines for HB were developed encompassing various studies. The guidelines indicated the limitations as a standardized treatment regimen could not be determined. The clinical guidelines proposed the use of acupuncture to treat HB based on previous evidence and noted that individualized treatment that considers the specific disposition and level and type of stress must be simultaneously provided [24]. Given the need for further research regarding individualized treatment, the present clinical study protocol was developed to use both fixed acupuncture points related to the major symptoms of HB, and variable acupuncture points based on traditional Korean medicine (TKM) principles.

Accordingly, we designed a randomized, sham-controlled, participant- and assessor-blinded, parallel-design pilot study using sham-treatment control and a 1:1 allocation. We will assess the clinical trial feasibility, and explore the effect and safety of acupuncture as a treatment for HB by comparing an experimental group (EG) receiving semi-individualized acupuncture with a control group (CG) receiving sham acupuncture.

## Method/design

### Study design

This study is a randomized, sham-controlled, participant- and assessor-blinded, parallel-design clinical trial. Participants will be assigned to the EG or the CG. The EG will be treated with real acupuncture, whereas the CG will be treated with sham acupuncture.

### Setting of the study

**Fig. 1:**
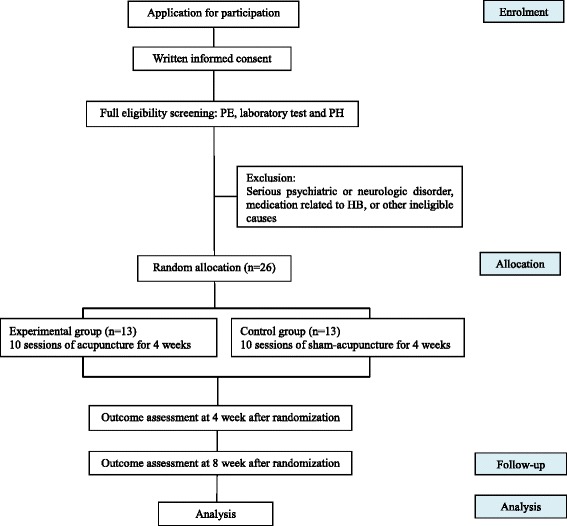
Trial flowchart

This clinical trial will be conducted at the outpatient department of a university hospital. Public recruitment advertising will be used to recruit participants to voluntarily apply to the trial, and a Korean medical doctor (KMD) will explain the details of the trial and will supply documented explanations. When the participants decide to enroll in the trial, they will be asked to submit written consent and to undergo evaluation to ensure compliance with inclusion/exclusion criteria. Participants who are eligible for the study will be randomly assigned to one of two groups and will receive the planned intervention and evaluation according to their corresponding group. Both groups will receive the intervention for a total of 10 sessions for 4 weeks (two to three sessions per week). A post-treatment assessment will be performed at 4 weeks after visit 1, and the study will be completed 8 weeks after visit 1 (Fig. 1).

### Recruitment

A participant recruitment document, including the study title, target participants, period, location and contents of the intervention, will be distributed as printed material, newspaper advertisements, and posters displayed at the clinical trial sites.

### Randomization and allocation concealment

An independent statistician will generate a table of random sampling numbers using Strategic Applications Software (SAS) ® Version 9.4 (SAS institute, Inc., Cary, NC, USA). The statistician will place the groups to be assigned into each double-layered, opaque envelope in sequence, seal the envelopes and write the numbers in order. The randomization code will be secured in a double-locked cabinet. As practitioners cannot be blinded by the nature of the intervention, the practitioners will assign the participants to one of the groups by opening the sealed envelopes in order in front of the participants. The opened envelope will be separately stored in a safe. The practitioner will confirm the eligibility before opening the envelope. If the participants do not meet the inclusion criteria, they will be removed from the study, and the randomization will not be performed (the envelope will not be opened). The reason for the lack of randomization will be recorded in a screening log and a Case Report Form (CRF).

### Sample size calculation

This study aims to evaluate clinical trial feasibility and to investigate basic information about effects and safety of acupuncture for the treatment of HB, rather than to satisfy hypothesis testing. Therefore, the sample size was not decided based on power calculations but based on the precision of mean and standard deviation (SD), the rationale of feasibility, and ethical issues that prohibit over-recruitment of participants. We ensured that the sample size exceeded the minimal number needed to assure the validity of the mean, SD and effect size and rationale of feasibility [25] and considered the study period and research budget [26, 27]. Accordingly, a sample size of 26 participants was estimated.

### Blinding of participants and assessors

As this study will use minimal acupuncture for the CG, the practitioners cannot be blinded; however, the participants and assessors will be blinded. Participants will be informed that they will be treated with either classical acupuncture or non-classical acupuncture [28]. Independent assessors who are not in charge of the intervention will ask only the questions necessary to evaluate and record in the CRF and will not know the assigned group throughout the study period. Un-blinding will be considered only under circumstances in which it is necessary for the rights and safety of the participants, including the occurrence of serious adverse effects. In such cases, the researchers will notify the principal investigator (PI) and document the subsequent processes.

## Characteristics of participants

### Inclusion criteria

1. Men and women who are aged 20 to 65 years
2. Meet the diagnostic criteria of the HBDIS: participants should have at least three of the four major symptoms (including chest discomfort, burning sensation of the face or chest, a surge of feeling, and sensation of a mass in the throat or in the epigastrium), one of two major psychological symptoms (including feelings of unfairness and mortification, and emotional resentment or ill will), two of four related somatic symptoms, and two of three related psychological symptoms. The symptoms should be related to stress, should cause psychosocial hypo-function, and should not be caused by a medical disorder
3. Provide written informed consent after being informed of the objectives and details of the clinical trial and agreeing to participate in the trial

### Exclusion criteria

1. History of serious psychiatric or neurologic disorder: hallucination, delusion, abuse of or dependence on alcohol or drugs, cerebrovascular disease, or degenerative disease of the central nervous system
2. Use of medications related to HB, such as anti-depressants or anti-anxiety, during the preceding 1 month
3. A seriously unstable medical condition: needing active medical treatment promptly, abnormal vital signs, or intolerable pain or poor food intake causing difficulty in activities in daily living (ADL)
4. Participation in other clinical trials
5. Female participants who are pregnant, breast-feeding, or planning to become pregnant
6. Residents of collective dwelling facilities, such as social welfare institutions
7. Failure to provide written informed consent
8. Lack of eligibility for the trial for other reasons: illiteracy, questionnaires are not understood or answered, or if the patient is not expected to be compliant with the treatment and evaluation visit schedule

## Intervention

### Intervention of experimental group

Ten acupuncture sessions will be conducted at fixed and individualized acupuncture points for 4 weeks by inserting an acupuncture needle perpendicular to the skin at a depth of 5.0–25.0 mm with manipulation for the *de-qi* and maintaining the position for 20 min each session. Disposable, sterile, 0.25 × 30-mm stainless steel acupuncture needles (Dongbang, South Korea) will be used. The fixed acupuncture points will be GV20, CV17, HT7, and ST36, and the individualized points will be decided according to the participants’ specific symptoms [24, 29, 30]. The locations of the acupuncture points are presented in the Standards for Reporting Interventions in Clinical Trials of Acupuncture (STRICTA) (Additional file 1). Qualified TKM doctors (KMD) with over 4 years of clinical experience will be in charge of the intervention.

### Treatment of control group

The CG will receive minimal acupuncture on non-acupuncture points 10 times over 4 weeks. The same type of acupuncture needle will be inserted perpendicular to the skin at a depth of 1.0–3.0 mm without manipulation for the *de-qi* and maintained for 20 min. The number of acupuncture points, duration, and frequency of the sessions will be the same as those for the EG, and the same practitioner will conduct the intervention.

- Location of non-acupuncture points:
  1. Upper extremities (UE) 1 (bilateral): 1 cm lateral from 5 cm below the midpoint of the transverse cubital crease
  2. Abdomen 1: 3 cm lateral from a point 13.5 cm above the umbilicus
  3. Lower extremities (LE) 1 (bilateral): 1.5 cm above EX-LE2 (the center of the upper border of the patella)
  4. LE 2 (bilateral): point at the upper one third of the medial part of the tibia
  5. LE 3 (bilateral): 1.5 cm below LE 2

### Allowed or prohibited concomitant treatment

All treatments for HB and symptom management will be prohibited. Other subjects’ treatments for underlying chronic diseases will be allowed if they do not change during the study period. If a violation occurs during the study period, the patient will be removed from the study.

## Outcome measure

### Feasibility outcomes

We will evaluate the feasibility of the study by assessing recruitment, compliance, and completion rates. Acceptability of acupuncture will be also evaluated by investigating effect and safety. The acupuncture treatment will be regarded as an acceptable intervention for a full-scale study if the effect size is greater than 0.2 in at least three of six major symptoms. With respect to safety, a full-scale study will be planned if there is no serious adverse event (SAE) associated with the intervention and if the incidence of adverse events (AEs) is not significantly different from the CG.

The integrity of the study will be examined as a qualitative indicator with regard to whether the study proceeds without any problems and whether a full-scale study is possible without modification of the pilot study design. This will be measured using the Acceptance Checklist for Clinical Effectiveness Pilot Trials (ACCEPT) [31]. We will check the blinding index to determine whether the blinding procedure requires modification. The blinding index ranges from − 1 to 1, with 0 being the most favorable blindness level [32].

### Clinical outcomes

Clinical outcomes include information regarding the possible effects and safety of acupuncture. The outcome measures regarding clinical effect will be assessed at baseline, week 3, week 5, and week 9. A Visual Analogue Scale (VAS) score for the four major somatic symptoms and two major psychological symptoms of HB will be the primary outcome measure. The major symptoms are chest discomfort, burning sensation of the face or chest, a surge of feeling, sensation of a mass in the throat or in the epigastrium, frequent feelings of unfairness and mortification, and emotional resentment or ill will.

The VAS is widely used for the assessment of variations in the severity of subjective symptoms. Participants mark their symptom severity on a 100-mm straight line whose extreme left end indicates “absence of symptoms” and whose extreme right end indicates “unbearably severe symptoms” [33, 34].

The secondary clinical outcome measures will be the VAS score for subjective anger, the BDI and the short-form of the Stress Response Inventory (SRI-short form). The IOME-HB and serum serotonin levels will be evaluated at baseline and week 5. The BDI is a self-reported inventory to evaluate the severity of depression with 21 questions, including somatic-affective and cognitive-affective factors. Each question is assigned one of 0 to 3 points, and the total score ranges from 0 to 63 [35, 36]. The SRI-short form is used to assess responses to stress. It contains 22 questions, including somatization factors (nine items), depression factors (eight items), and anger factors (five items) [37]. The IOME-HB is used to evaluate the severity of psychiatric and somatic symptoms of each of five types of HB, according to TKM principles, by participants scoring their symptoms using a 5-point Likert scale [4, 5]. Serum serotonin levels will be measured by analyzing the blood drawn from the veins of the arm. We will quantitatively analyze the numbers themselves to see whether there are differences before and after treatment and between groups.

**Fig. 2:**
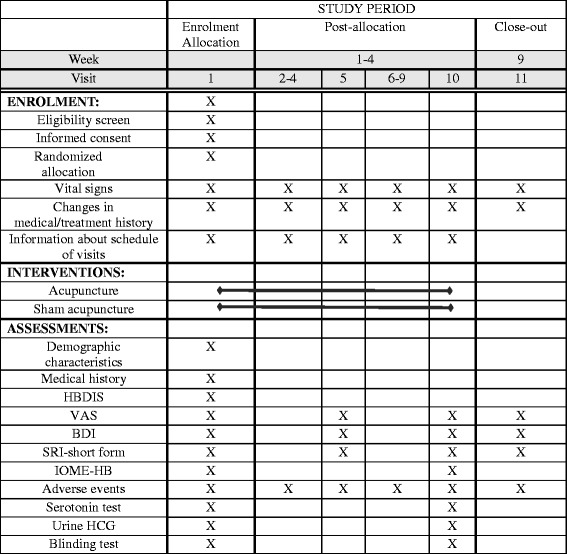
The Standard Protocol Items: Recommendations for Interventional Trials (SPIRIT) Figure. The schedule of enrollment, interventions, and assessments

The follow-up evaluation will be performed 4 weeks after the completion of the treatment and will include the VAS for core symptoms, VAS for subjective anger, and the BDI and SRI-short form. To remind participants and encourage their participation in the follow-up assessment, we will inform participants about their visits by phone 1 to 7 days prior to the follow-up visit. Adverse events (AEs) will be recorded at every visit to assess the safety of the trial. The Standard Protocol Items: Recommendations for Interventional Trials (SPIRIT) schedule of enrollment, interventions, and assessments is shown in Fig. 2. The SPIRIT Checklist can be found in Additional file 2.

### Criteria for dropout

1. The subject or the legal representative of the subject withdraws their participation
2. A violation of the inclusion/exclusion criteria is found during the course of the clinical trial
3. The development of an SAE or an AE causes difficulty in continuing
4. Less than 80% compliance with the treatment
5. The subject violates the plan of the clinical trial
6. Failure to track the subject
7. The subject takes medications that affect the results during the study period

### Data collection, management, and personal information protection

Data will be collected by the KMD who will be in charge of the questionnaire and evaluation. The KMD will have six or more years of specialized education and four or more years of clinical experience and will complete a workshop on evaluation standardization. Soft copies will be saved on a storage device not connected to the Internet. All data will be kept in locked storage, and only those with permission from the PI will be able to access the data. All blood samples will be disposed of immediately after they are analyzed. All retained data will contain codes to identify patients instead of personal information.

### Statistical analysis

The recruitment rate, compliance rate, and completion rate will be presented as a number and percentage and will also be presented for each group. For clinical outcomes, including VAS scores for the core symptoms of HB and subjective anger, the BDI, the SRI-short form and serum serotonin levels, the mean SD at each assessment point and Cohen’s *d*, which is an estimated effect size that indicates the standardized difference between two mean values, will be presented [38]. Effect size will be calculated based on the value immediately after the end of the 4-week treatment (visit 10).

Analyses will mainly be performed using the full analysis set (FAS) population, while the per-protocol set (PPS) population will be analyzed as a supplement. Although the intention-to-treat (ITT) ideal includes all randomized subjects, the FAS approach acknowledges the practical infeasibility of completely following all participants until study completion and includes as complete and as similar a population to ITT as possible [39]. The FAS population does not include the following: (1) individuals who do not meet the inclusion criteria, (2) individuals who have never undergone acupuncture treatment, and (3) individuals who have never been assessed since randomization and, therefore, have no data available for collection. The PPS population will include the following: (1) individuals for whom acupuncture accounts for more than 80% of the total number of treatments received, (2) individuals who have had their evaluation parameters measured, and (3) individuals who do not violate the clinical trial protocol [40]. Missing data will be replaced by multiple imputation. Statistical analyses will be conducted by an independent statistician using SAS® version 9.4 (SAS Institute Inc., Cary, NC, USA).

### Monitoring

The clinical study will be regularly monitored by independent researchers who are specifically responsible for monitoring. They will ensure that the rights and welfare of the study participants are protected, confirm the accuracy of the data, and maintain the safety and verifiability of the study. They will also verify that the clinical study complies with the study protocol and related regulations. At each monitoring visit, the researchers will confirm and store data, such as original patient records, treatment records, and storage status. Any problems observed during the course of the study will be recorded and reported. Corrective measures will be taken accordingly. If the problems are serious and compromise the safety and validity of the study, they will be reported to the Institutional Review Board (IRB), which will determine whether the clinical study can continue.

### Safety issues

AEs refer to all harmful and unintended reactions that occur during the acupuncture treatment. The possible adverse effects of acupuncture include the following: local bleeding or haematoma, more than slight sharp pain, aggravation of symptoms, drowsiness, syncope, nausea, headache, seizure, peripheral nerve injury, and infection. The participants will be informed of possible AEs in advance.

Participants will be questioned about recent hospital visits, new medications taken or new symptoms arising, at every visit. The symptoms, date of occurrence, disappearance, causality, and severity of the AEs will be recorded, and the severity will be recorded using the three-level classification by Spilker et al. [41].

During the study period, all researchers will attempt to ensure patient safety. When SAEs occur, the researchers will immediately halt the study and promptly provide appropriate management. The PI will also report adverse symptoms to the IRB and research clients and halt the study partially or completely until further notice.

## Discussion

HB, which literally means anger syndrome, leads to both somatic and psychological symptoms. The psychological symptoms mainly include an inadequate expression of anger and trait anger, and the somatic symptoms mainly include palpitations, chest discomfort and gastrointestinal disorders [7, 42, 43]. Regarding the causes of HB, a person’s specific disposition, stress from a difficult marital relationship or a serious life crisis can both act as risk factors of HB; enduring negative emotions for a long period of time is known to be a root cause of HB [44].

Patients with HB tend to avoid psychiatric treatment and to rely on treatments that focus only on their physical symptoms [43, 45]. These patients also receive insufficient social support [42]. Despite relatively high levels of self-awareness of HB among these patients [3, 10, 11], they have been unable to receive effective treatment; therefore, further research is needed to develop more effective and acceptable treatments for HB.

This is a pilot study that aims to evaluate the feasibility of acupuncture treatment for HB and to confirm basic information about its effects and safety [46]. In pilot studies, determining feasibility should be the first priority, and data regarding treatment effects should be descriptively analyzed to avoid exaggerated interpretations [47–51]. Therefore, this trial primarily aims to obtain information about the recruitment, consent, and integrity of the study protocol based on recruitment rate, compliance rate, and completion rate. Clinical effects will be assessed to explore the effects and safety of the intervention and to determine the effect size [47, 52, 53]. We will present Cohen’s *d*, which provides an estimated effect size, because the effect size provides numerical information about the actual difference between two outcomes whereas *p* values are affected by sample size and only provide a dichotomous assessment of statistical significance [54–57]. These data will provide the information needed to calculate the sample size as well as information on the intervention effects that can be easily compared to interventions applied in other studies [38, 47].

With respect to the inclusion/exclusion criteria, an age range of 20 to 65 years was selected to cover as wide a range as possible while at the same time protecting those who are underage or elderly. In addition, the exclusion of participants taking medication related to HB is essential to obtain accurate results of clinical studies, and is determined by considering the possibility of recruitment and ethical factors. As many HB patients tend not to visit hospitals and take medications for somatic symptoms only [43, 45], we judged that recruiting participants would not have serious ethical problems because it is presumed not to interrupt their proper treatments. Moreover, HB is not a life-threatening disease but reflects a chronic process. There are no specific drugs for HB, and we thus concluded that the prohibition of concurrent treatment during the total 8-week study period would not cause serious problems. Those who reside in collective dwelling facilities are excluded because they are prohibited from being subjects in clinical trials by Korean law in order to protect their human rights.

For the acupuncture points, GV20, CV17, and ST36 were selected from the points that are traditionally widely used and known to be effective based on previous research [29] textbook information [30], and guidelines [24]. This selection excluded the points that could cause severe pain or discomfort during the procedure. HT7 was selected based on a previous study that revealed positive effects on HB [23] and serotonin regulation [58].

The use of a CG is known to be reasonable and necessary to provide information that is as accurate as possible for future research [46, 59]. Therefore, we designed this pilot study with a CG to obtain realistic information under a setting similar to those used in RCTs. The CG will receive shallow acupuncture at non-acupuncture points in order to mimic true acupuncture treatment and minimize the therapeutic effects [20–22]. The non-acupuncture points are located far from the true acupuncture points to compensate for the limitations of the previous studies [20–22] and to minimize the non-specific effects of sham acupuncture [60, 61]. To assess the appropriateness of this research design, we will analyze the results of the blinding index. Accordingly, we will obtain information that will help determine the appropriate type of CG to be used in future research.

To confirm the selection of the most appropriate primary outcome measures and possible objective outcome measures, the BDI, the SRI-short form, and serum serotonin levels will be measured. HB is commonly accompanied by one or more psychiatric disorders and exhibits unique symptoms. Therefore, consistent research on the correlation between HB and other DSM diagnoses is necessary. In this study, we will explore the correlation between the symptoms of HB and other psychiatric disorders by evaluating the BDI and SRI-short form [7, 62]. Serum serotonin levels will be experimentally assessed to evaluate possible correlations between the mechanism of acupuncture’s effects on HB and serotonin and to assess the appropriateness of the evaluation in future RCTs [13, 58, 63–67]. Previous studies on HB and serotonin have demonstrated that the central serotonin system is related to anger, depression, and anxiety and that serotonin is involved in the behavioral patterns related to the feeling of injustice, a key appraiser in HB [68, 69]. In an animal study, acupuncture on HT7 has been reported to modulate the serotonin system in depression-related behaviors [58].

HB is listed as a culture-bound syndrome in Korea, and there are no reports of global prevalence. However, approximately 7000 to 23,000 people have immigrated abroad over the past decade [70], and approximately 1.7 million immigrants to the United States account for a high percentage of Asian Americans [71]. Moreover, a study conducted on Korean Americans revealed that 12% of them have HB [11]. In addition, considering the cumulative stress due to unfair treatment and injustice with respect to the cause of HB, there are likely to be similar subjects in countries with similar social conditions. It is possible that these patients are being treated only for individual physical symptoms not identified as HB [43, 45].

This study has some limitations. First, this clinical trial will include a small population of participants and will not primarily aim to perform hypothesis testing. Therefore, the results of this study cannot be generalized as basic data for evaluating the effect and safety of acupuncture for HB. Moreover, because the CG will receive minimal acupuncture, we cannot neglect the possibility of physiological activation caused by needle penetration. The effect of acupuncture will need to be carefully interpreted because the acupuncture points, needling depth, and *de-qi* will be the only differences between groups.

However, as this trial assesses the use of optimized, semi-individualized acupuncture for HB to enable the design of larger RCTs, we expect that it can provide feasibility data and basic information about the effect and safety of acupuncture for HB.

### Trial status

This clinical trial is currently in the recruiting phase.

## Additional files

Additional file 1: Checklist of Standards for Reporting Interventions in Clinical Trials of Acupuncture (STRICTA) (DOCX 19 kb)

Additional file 2: SPIRIT 2013 Checklist: recommended items to address in a clinical trial protocol and related documents. (DOC 121 kb)

## Abbreviations

BDI: Korean version of the Beck Depression Inventory
CG: Control group
CRF: Case Report Form
DSM: *Diagnostic and Statistical Manual of Mental Disorders*
EG: Experimental group
FAS: Full analysis set
GAD: Generalized anxiety disorder
HB: *Hwa-byung*
HBDIS: *Hwa-byung* Diagnostic Interview Schedule
IOME-HB: Instrument of Oriental Medical Evaluation for HB
IRB: Institutional Review Board
ITT: Intention-to-treat
KMD: Traditional Korean medical doctor
LE: Lower extremities
MDD: Major depressive disorder
PI: Principal investigator
PPS: Per-protocol set
SRI-short form: Short form of the Stress Response Inventory
TKM: Traditional Korean medicine
UE: Upper extremities
VAS: Visual Analogue Scale.
