# Supplementary material for: Effect and safety of acupuncture for Hwa-byung, an anger syndrome: a study protocol of a randomized controlled pilot trial
Source: Trials. 2018 Feb 9;19:98. doi: 10.1186/s13063-017-2399-0 (PMC5807845; doi:10.1186/s13063-017-2399-0)
Supplement: Supplementary file 1 — Checklist of Standards for Reporting Interventions in Clinical Trials of Acupuncture (STRICTA) (DOCX 19 kb) [file 13063_2017_2399_MOESM1_ESM.docx]

Additional file 1. Checklist of Standards for Reporting Interventions in Clinical Trials of Acupuncture (STRICTA)

| **Item** | **Detail** | **Contents** |
| --- | --- | --- |
| **Acupuncture rationale** | Style of acupuncture | Traditional Korean medical body acupuncture |
|  | Reasoning for treatment provided based on historical context, the literature, and consensus methods, with references where appropriate | Consensus of three TKM experts, textbooks[30], clinical guidelines[24] and previous studies[23, 29]. |
|  | Extent to which treatment varied | Semi-individualized |
| **Details of needling** | Number of needle insertions per subject per session | 10 points |
|  | Names (or location if no standard name) of points used (uni/bilateral) | 1) Fixed points: GV20, CV17, HT7 (bilateral), ST36 (bilateral)   1. GV20: On the head, 5 cun* superior to the anterior hairline, on the anterior median line. 2. CV17: In the anterior thoracic region, at the same level as the 4th intercostal space, on the anterior median line. 3. HT7: On the anteromedial aspect of the wrist, radial to the flexor carpi ulnaris tendon, on the palmar wrist crease.   (4) ST36: On the anterior aspect of the leg, on the line connecting ST35 with ST41, 3 cun inferior to ST35.  2) Selective points: two individualized points according to individual symptoms |
|  | Depth of insertion based on a specified unit of measurement or on a particular tissue level | 5.0-25.0 mm |
|  | Response sought (e.g., De-qi or muscle twitch response) | De-qi including soreness, swelling or numbness |
|  | Needle stimulation (e.g., manual, electrical) | Manual acupuncture |
|  | Needle retention time | 20 minutes |
|  | Needle type (diameter, length, and manufacturer or material) | Sterile acupuncture needle with 0.25x30-mm stainless steel (Dongbang, South Korea) |
| **Treatment regimen** | Number of treatment sessions | 10 sessions |
|  | Frequency and duration of treatment sessions | For 4 weeks (2 to 3 sessions per week) |
| **Other components of treatment** | Details of other interventions administered to the acupuncture group (e.g., moxibustion, cupping, herbs, exercises, lifestyle advice) | Psychotropic drugs and TKM treatments related to HB will be prohibited |
|  | Setting and context of treatment, including instructions to practitioners and information and explanations provided to patients | Practitioners can talk to the participants about topics necessary to the treatment only, not about other irrelevant topics |
| **Practitioner background** | Description of participating acupuncturists (qualification or professional affiliation, years in acupuncture practice, other relevant experience) | KMDs with over 4 years of clinical experience |
| **Control or comparator interventions** | Rationale for the control or comparator in the context of the research question with sources that justify this choice | Shallow acupuncture insertion on non-acupuncture points has been used in previous clinical trials on acupuncture for HB[20-22]. |
|  | Precise description of the control or comparator. If sham acupuncture or any other type of acupuncture-like control is used, provide details as for Items 1 to 3 above | Sham acupuncture will consist of shallow acupuncture insertion (under 3 mm in depth) on non-acupuncture points and will not arouse de-qi |

TKM, traditional Korean medicine; *cun, 1.5 cm; KMD, traditional Korean medical doctor; HB, Hwa-byung
